# Is There a Processing Preference for Object Relative Clauses in Chinese? Evidence From ERPs

**DOI:** 10.3389/fpsyg.2018.00995

**Published:** 2018-07-09

**Authors:** Talat Bulut, Shih-Kuen Cheng, Kun-Yu Xu, Daisy L. Hung, Denise H. Wu

**Affiliations:** ^1^Institute of Cognitive Neuroscience, National Central University, Taoyuan City, Taiwan; ^2^Department of Speech and Language Therapy, Istanbul Medipol University, Istanbul, Turkey; ^3^College of Humanities and Social Sciences, Taipei Medical University, Taipei, Taiwan

**Keywords:** relative clauses, Chinese sentence comprehension, working memory, integration resources, storage resources, linear distance, structural distance, event-related potentials

## Abstract

A consistent finding across head-initial languages, such as English, is that subject relative clauses (SRCs) are easier to comprehend than object relative clauses (ORCs). However, several studies in Mandarin Chinese, a head-final language, revealed the opposite pattern, which might be modulated by working memory (WM) as suggested by recent results from self-paced reading performance. In the present study, event-related potentials (ERPs) were recorded when participants with high and low WM spans (measured by forward digit span and operation span tests) read Chinese ORCs and SRCs. The results revealed an N400-P600 complex elicited by ORCs on the relativizer, whose magnitude was modulated by the WM span. On the other hand, a P600 effect was elicited by SRCs on the head noun, whose magnitude was not affected by the WM span. These findings paint a complex picture of relative clause processing in Chinese such that opposing factors involving structural ambiguities and integration of filler-gap dependencies influence processing dynamics in Chinese relative clauses.

## Introduction

There has been a great deal of interest in relative clauses (RCs) in psycholinguistic research. This interest stemmed mainly from the complexity and non-canonicity of RCs, which afforded empirical tests of psycholinguistic postulates. For instance, RCs were used to test the psychological reality of traces and gaps (McElree and Bever, 1989), such as *t*_*i*_ in (1a) and (1b). Also, since the moved head noun (*the scientist* in 1a and 1b) must be carried unattached while the intervening material is processed (Traxler et al., 2002), this was used to obtain insights into the role of verbal working memory (WM) in language processing (Just and Carpenter, 1992). Importantly, as the English subject and object RCs differed minimally in surface form [only the ordering of the noun phrase (NP) and the verb phrase (VP)] but differed substantially in terms of structure (notice the locus of *t* in 1a and 1b), they have been found to be apt for experimental study. The general finding in this line of research is that subject relative clauses (SRCs) are processed more easily than object relative clauses (ORCs). The same finding was reported for different languages including English (King and Kutas, 1995; Traxler et al., 2002), Dutch (Frazier, 1987a), French (Cohen and Mehler, 1996), German (Mecklinger et al., 1995) and Spanish (Betancort et al., 2009).

1a. Subject Relative Clause:
  The scientist_i_ who *t*_i_ praised the author smiled.
1b. Object Relative Clause:
  The scientist_i_ who the author praised *t*_i_ smiled.

To test the cross-linguistic universality of SRC advantage, some studies were carried out in typologically different languages such as Chinese (Hsiao and Gibson, 2003), Japanese (Ishizuka, 2005), Korean (Kwon et al., 2006), and Basque (Carreiras et al., 2010). However, these studies produced conflicting results, with some confirming SRC advantage (Kwon et al., 2006), while some others finding ORC advantage (Hsiao and Gibson, 2003; Carreiras et al., 2010). Therefore, further studies are needed in typologically different languages such as Chinese to test the asserted universality of SRC processing advantage.

The current study aimed to shed light on this controversial issue by recording event-related potentials (ERPs) while the participants read Chinese SRCs and ORCs. Furthermore, the potential effect of individual WM span on RC processing patterns was also investigated by conducting two WM span tests. The following sections describe the RC processing accounts, previous studies on Chinese RCs, and the ERP components of interest.

### Accounts of relative clause processing

The major theories of RC processing are presented below with their predictions for English and Chinese RCs.

#### Structural distance hypothesis

Certain theories claim that the underlying syntactic structure is uniform across languages and, therefore, they argue for universal processing dynamics in certain aspects of sentence processing. One of the prominent accounts in this category is the Structural Distance Hypothesis (SDH, O'Grady et al., 2003). In this approach, structural distance refers to the amount of syntactic nodes/projections in the syntactic tree that intervene between the head noun and the gap. In SRCs, as in (2a), the gap position *e*, from which the head noun is extracted, is within the inflection phrase (IP). In ORCs, as in (2b), the gap is embedded in the VP, which is deeper than the IP in the syntactic structure. This hierarchical ordering of subject and object positions is assumed by almost all theories of syntax (O'Grady et al., 2003). In other words, there are more syntactic nodes intervening between the gap and the head in ORCs than in SRCs (Collins, 1994), and this holds true for both head-initial languages such as English and head-final languages such as Chinese (Carreiras et al., 2010). Therefore, SDH predicts ORC disadvantage in any language.

2a. The scientist_i_ [CP who_i_ [IP e_i_ [VP praised the author]]] smiled.
2b. The scientist_i_ [CP who_i_ [IP the author [VP praised e_i_]]] smiled.
  [CP: Complementizer phrase; IP: Inflectional phrase; VP: Verb phrase]

#### Memory-based accounts

Memory-based accounts claim that sentence processing is limited by WM capacity. Therefore, if there are elements in a sentence that occupy WM space, this may lead to processing difficulties. Gibson's Dependency Locality Theory (DLT; Gibson, 1998, 2000) is an example of a memory-based account. According to DLT, there are two metrics, namely, storage resources and integration resources, which can account for sentence processing dynamics.

##### Storage Resources

According to the metric of storage resources, successful processing of a sentence requires keeping track of incomplete head-dependencies (Hsiao and Gibson, 2003). The ORC difficulty in English is explained with reference to the higher number of temporarily incomplete dependencies in ORCs than SRCs. For instance, if we compare the SRC and ORC in (1), we see that at the location where the two RCs start to differ (*praised* and *the*), the number of incomplete dependencies varies between the RCs. Specifically, in the SRC, after reading the part of the sentence “The scientist who praised,” only two heads are needed: a noun as the object of the RC and a verb for the matrix sentence. However, in the ORC, after reading “The scientist who the,” three syntactic heads are required: a noun for the determiner “the,” a verb for the RC, and a verb for the matrix sentence. Thus, the difficulty associated with ORCs can be explained by the storage of greater number of incomplete head-dependencies.

On the other hand, in Chinese object-modifying RCs as in (3), SRCs and ORCs have the same structure until the relative clause NP/VP that follows the matrix verb; therefore, until this region processing patterns should be similar. When the verb *beat* in (3a) is encountered, however, the reader realizes that an RC is being read, since a verb follows another verb instead of an NP that would serve as the object. Therefore, three syntactic heads are required: two NPs as the RC object and subject (the latter also functioning as the object of the matrix verb) and an RC marker. On the next word *star*, two syntactic heads are needed: an RC marker and a subject NP for the RC verb. On the relativizer *de*, only an NP is predicted as the subject of the RC.

3a. Object-Modifying Subject Relative Clause:
  民眾討厭那個毆打明星的攝影師
people hate the e_i_ beat star *de* photographer_i_
People hate the photographer who beat the star.
3b. Object-Modifying Object Relative Clause:
民眾討厭那個明星毆打的攝影師

people hate the star beat e_i_ *de* photographer_i_ People hate the photographer who the star beat. (*e* refers to empty category or the gap site)

As for the ORC in (3b), when reading the embedded noun *star*, no head is predicted since this NP can conclude the sentence by serving as the object NP for the matrix clause. However, on the next word, *beat*, a matrix clause reading is no longer tenable, leading the reader to entertain an RC reading or a subordinate clause reading (such as a that-clause: People hate (that) the photographer beat the star). If an RC reading is adopted by the reader, two heads are predicted on the embedded verb *beat*: an RC marker and an NP as the object of the RC (which also functions as the object of the matrix verb). If a subordinate clause reading is entertained, only one head is predicted on this verb: an object NP. On the following word, the relativizer *de*, as in the SRC, the RC reading becomes obvious and only an NP is predicted to serve as the object of the RC. Therefore, the storage resources metric predicts ORC advantage for Chinese due to the smaller number of predicted syntactic heads on the embedded ORC noun and verb, unlike its prediction of SRC advantage for English.

##### Integration Resources

The metric of integration resources capitalizes on the process of establishing connection between the incoming word and the current syntactic structure (Gibson, 1998, 2000). Integration cost is calculated by the distance of the dependency (Hsiao and Gibson, 2003). For instance, in the SRC in (1a), the wh-filler *who* and the verb *praised* are connected locally, without any intervening material. However, in the ORC in (1b), the NP *the author* separates the wh-filler and the RC verb, hence more distant dependency. This account has also been conceptualized as the Linear Distance Hypothesis (LDH) (Carreiras et al., 2010) due to its emphasis on the linear, rather than structural, distance of dependencies. While the metric of integration resources predicts SRC advantage for English, it predicts ORC advantage for Chinese because the head noun [*photographer* in (3)] and the RC verb [*beat* in (3)] needs to be integrated and this integration is more local in Chinese ORCs as in (3b) with fewer intervening elements than in SRCs.

##### Similarity-Based interference

In addition to the two memory-based approaches proposed by DLT, similarity-based interference is another account that highlights the role of WM in sentence processing (Gordon et al., 2001). Proponents of this account quantify the effect of WM on processing dependencies in terms of similarity of the items in memory (Gordon et al., 2001; Lewis and Vasishth, 2005; Van Dyke and McElree, 2006). For instance, Gordon et al. (2001) found that when the second NP in RCs [such as *the author* in (1)] was replaced with a pronoun (e.g., *you*) or a name (e.g., *Joe*), the difference between SRCs and ORCs was reduced or eliminated. The interference is reduced when the two referents in RCs are of different types, hence the cues associated with them do not interfere with one another. This interference effect might occur during the encoding, storage or retrieval of the NPs.

For the sentences of (3a) and (3b), the same degree of similarity-based interference between the two descriptive NPs [e.g., *star* and *photographer* in (3)] would be predicted to occur in SRCs and ORCs due to the same type of referents used. However, theories emphasizing decay and interference (Lewis and Vasishth, 2005; Vasishth et al., 2013) would predict more difficulty with SRCs because the object NP [*star* in (3a)] intervenes between the RC verb and the subject NP, potentially causing interference. In addition, decay of the referent is greater in SRCs than ORCs due to the longer linear distance between the filler-gap dependency in the former than the latter sentence construction.

#### Frequency-based accounts

Corpus studies and psycholinguistic research have shown that frequency of linguistic structures affects processing dynamics (Reali and Christiansen, 2007). Based on such findings, frequency-based accounts argue that the more frequent a particular structure is in a certain language, the easier its processing will be. In some theories of this approach, the notion of frequency has been formalized as experience and surprisal (Hale, 2001; Levy, 2008) or entropy (Hale, 2003). Consistent with such reasoning, constraint-based accounts assert that alternative structural interpretations of a sentence being processed are partially activated based on frequency, plausibility and other constraints (McRae et al., 1998; Gennari and MacDonald, 2008). Accordingly, comprehension difficulty results from the competition between partially activated alternative structures depending on the linguistic experience of the listeners/readers.

Analyses of various spoken and written corpora revealed that SRCs were overwhelmingly more frequent than ORCs in English, which is consistent with processing difficulty associated with ORCs (Roland et al., 2007). Furthermore, it was found that the majority of ORCs in English corpora occurred as pronominal RCs compared to SRCs (Reali and Christiansen, 2007). In other words, the noun in ORCs was usually a pronoun (e.g., *you*) rather than a full NP (e.g., *the lawyer*), as exemplified in this ORC: “The barber that *the lawyer*/*you* admired”. Consistently, it was shown through self-paced reading studies that ORCs with embedded personal pronouns were actually read faster than SRCs (Reali and Christiansen, 2007). In summary, the parallel results between empirical reading performance and distributional patterns observed in corpora support the idea that structural frequency plays a key role in processing patterns of English RCs. Similarly, the frequency-based accounts also predict SRC advantage for Chinese, as previous corpus studies generally indicated that SRCs are more frequent than ORCs in Chinese (Hsiao and Gibson, 2003; Pu, 2007; Vasishth et al., 2013), as well.

In summary, the major accounts of RC processing make divergent predictions about processing patterns of Chinese RCs, providing an opportunity to put these accounts to empirical test, which is not afforded by English RCs as outlined above. However, it should be cautioned that RCs in English as well as other Indo-European languages exhibit an importance difference from those in Chinese, which may complicate their comparison. Specifically, Chinese RCs are prenominal; i.e., the relative clause noun and verb (e.g., *star beat* in 3b) precede the modified head noun (e.g., *photographer*), unlike the post-nominal English relative clauses (e.g., *the photographer who the star beat*). Furthermore, there is no morpheme or inflection that signals the existence of a relative clause, except for the relative clause particle (i.e., relativizer *de*), which marks a relative clause construction. Therefore, as pointed out by the editor of the current paper, this RC configuration in Chinese results in a processing pattern where the reader does not unambiguously parse the current structure as a relative clause at least until the relativizer is reached, and may entertain other plausible parses (e.g., a main clause reading: *the star beat the photographer*) until then. Furthermore, even when the relativizer is finally reached, there is still no competition between Chinese SRCs and ORCs, as the configuration of the preceding RC verb and noun already resolve which RC type is being read. In contrast, RCs in Indo-European languages involve garden-paths, and much psycholinguistic research has employed such RCs to investigate which structure (SRC vs. ORC), if not both, is entertained in case of temporal ambiguity and which factors affect the parsing preferences (e.g., semantic and morphological information, or working memory span) (Frazier, 1987b; Mecklinger et al., 1995; Friederici et al., 1998). These studies conducted in languages with verb/auxiliary final constructions and scrambling such as German, Dutch and Italian employed RCs that would be disambiguated only at the sentence-final verb/auxiliary, and demonstrated SRC preference.

Taken together, these points suggest that in Chinese, SRCs and ORCs do not compete as alternative structures as they do in English, where the two structures exhibit temporal ambiguity immediately at onset (the photographer who *the star beat/beat the star)*. Hence, relative clause processing in Chinese is qualitatively different from that in English and other Indo-European languages with post-nominal relative clauses; i.e., it is not clearly possible to investigate ambiguity-related parsing preferences in subject and object relative clauses. Although there is no temporal ambiguity between the RC types in Chinese, this configuration enables examination of the effects of linear distance (number of intervening elements) and the structural distance (number of syntactic projections) between the gap and the filler, especially after encountering the relativizer. In particular, the head noun is the site where the filler-gap integration occurs; in other words, the head noun is integrated with its verb phrase, rendering it the most ideal site to test the putative processing clause asymmetry between the RC types in Chinese. Furthermore, as pointed out above, the frequency of SRCs is higher than ORCs in Chinese, which may also affect processing patterns after an RC parse is unambiguously entertained (i.e., on the relativizer and the head noun). Hence, Chinese RCs provide a means to test the Structural Distance, Linear Distance and Frequency-based Accounts.

### Previous studies on chinese relative clauses

All theories of RC processing predict SRC advantage for English. However, whereas the structural and frequency-based accounts predict SRC advantage for Chinese, the memory-based account predicts ORC advantage. A number of studies were conducted in Chinese to test these accounts, as summarized in Table 1 comprehensively.

**Table 1:** Overview of previous studies comparing Chinese subject and object relative clauses.

**Method**	**Study**	**Modified matrix position**	**Results**			**Advantage**	**Theories supported**
			**Embedded N & V**	**Relativizer**	**Head noun**		
ERPs	Packard et al., 2010	Subject	ORC>SRC (larger N400 in ORC)	SRC>ORC (larger P600 in SRC)	—	ORC	Integration resources
		Object	ORC>SRC^a^ (larger P600 in ORC)	—	SRC>ORC (larger P600 in SRC)		
	Yang and Perfetti, 2006	Subject	SRC>ORC (larger anterior SN in SRC) ORC>SRC (larger posterior SN in ORC)	—	—	ORC^b^	Multiple factors including integration & storage resources
		Object	SRC>ORC (larger N400, P600 & anterior SN in SRC) ORC>SRC (larger N400 in ORC)	—	—		
	Yang et al., 2010	Object	SRC>ORC (larger N400 & P600 in SRC) ORC>SRC (larger N400 in ORC)	—	SRC> ORC (larger right-lateralized SN in SRC) ORC>SRC (larger central-frontal SN in ORC)	ORC^b^	Multiple factors including integration resources
Self-paced reading	Chen et al., 2008	Subject	SRC>ORC	—	—	ORC^c^	Storage resources
	Gibson and Wu, 2013	Subject	—	—	SRC>ORC	ORC	Integration resources
	He and Chen, 2013	Subject	SRC>ORC (animate-inanimate)	SRC>ORC (animate-inanimate)	SRC>ORC (animate-inanimate) ORC>SRC (inanimate-animate)	ORC^d^	Thematic fit & frequency-based account
		Object	SRC>ORC (animate-inanimate & inanimate-animate)	SRC>ORC (animate-inanimate)	SRC>ORC (animate-inanimate)	ORC	
	Hsiao and Gibson, 2003	Subject	SRC>ORC	—	—	ORC	Storage resources; word order canonicity
	Jäger et al., 2015	Subject	ORC>SRC	—	—	SRC	Expectation-based account
		Object	ORC>SRC^a^	—	—	SRC	
	Lin, 2006	Subject	—	ORC>SRC	ORC>SRC	SRC	Structural distance hypothesis
		Object	—	ORC>SRC	ORC>SRC		
	Lin and Garnsey, 2011	Object	SRC>ORC	SRC>ORC	SRC>ORC^a^	ORC	Storage resources; integration resources; word-order canonicity; similarity-based interference
	Lin, 2014	Subject	SRC>ORC	—	—	ORC	Storage resources^e^
	Li et al., 2010	Subject	—	—	ORC>SRC	SRC	Frequency-based account
		Object	—	—	ORC>SRC		
	Vasishth et al., 2013	Subject	—	SRC>ORC	ORC>SRC	SRC^f^	Expectation-based account
	Wu et al., 2012	Subject	ORC>SRC	ORC>SRC	ORC>SRC	SRC^g^	Constraint-satisfaction model
Eye-tracking	Jäger et al., 2015	Subject	ORC>SRC	ORC>SRC^a^	—	SRC	Expectation-based account
		Object	ORC>SRC	—	ORC>SRC^a^	SRC	
	Sung et al., 2015	Subject	ORC>SRC^h^ & SRC>ORC	—	SRC>ORC	ORC	Storage resources; integration resources; word order canonicity; perspective shift
	Sung et al., 2016	Subject	ORC>SRC	—	SRC>ORC	ORC	Linear distance hypothesis
Maze task	Qiao et al., 2012	Subject	SRC>ORC	ORC>SRC	—	ORC	Expectation-based account; storage resources
Modeling	Hsiao and MacDonald, 2013	Subject	ORC>SRC	—	SRC>ORC	ORC	Experience-based account
		Object	SRC>ORC & ORC>SRC	—	ORC>SRC	SRC	
	Chen et al., 2012^i^	Subject	ORC>SRC & SRC>ORC	ORC>SRC	—	SRC	Frequency/experience-based account
		Object	ORC>SRC & SRC>ORC	—	—		
Production by children	Hsu et al., 2009	Subject	ORC>SRC^j^			SRC	Structural distance hypothesis
		Object					
Aphasia	Law and Leung, 2000	Subject	SRC>ORC			ORC	Linear/canonical order
	Law, 2000^k^	Subject	SRC>ORC			ORC	Linear/canonical order
	Su et al., 2007	Subject	SRC >ORC			ORC	Trace deletion hypothesis

“>” indicates greater processing difficulty reflected by slower RTs, larger ERP effects, etc.; N, Noun; ORC, Object relative clause; SN, Sustained negativity; SRC, Subject relative clause; V, Verb.

a Marginally significant.

b ORC preference here is not explicitly articulated in the relevant article, but based on the present paper's author's interpretation of the N400-P600 complex found for SRC.

c *Only for low working memory span readers*.

d *ORC preference with animate-inanimate NP configuration; SRC preference with inanimate-animate NP configuration*.

e *The author argues for complex effects of various factors including thematic order canonicity and consistency, depending on the regions of interest*.

f *SRC preference in Experiment 1 and 2, ORC preference in Experiment 3*.

g *No difference between SRCs and ORCs when the relative clause subject was animate and the object was inanimate*.

h *Significant only in by-items analysis*.

i *No statistics were reported*.

j *ORC was produced less frequently and more erroneously than SRC*.

k *This paper examines performance patterns of Chinese aphasics from three previous studies (Su and Law, 1993; Law and Leung, 1998, 2000)*.

One of the first studies on RC processing in Chinese was conducted by Hsiao and Gibson (2003), who compared processing differences between SRCs and ORCs in a self-paced reading paradigm to determine whether native speakers of Mandarin Chinese exhibited SRC advantage or disadvantage. In contrast with previous findings from English and other languages (Cohen and Mehler, 1996; Traxler et al., 2002; Kwon et al., 2006). Hsiao and Gibson (2003) reported better comprehension and shorter response times for ORCs than SRCs in Chinese, demonstrating ORC advantage. They interpreted their findings to support the storage resources metric. This seminal study spawned a number of studies on Chinese RCs as summarized below.

#### Previous studies reporting SRC advantage

Some of the previous self-paced reading studies reported shorter reading times in SRCs than ORCs in Chinese, and interpreted this as SRC advantage in support of variants of the Structural Distance Hypothesis assuming universal processing difficulty with extraction from the object position compared to the subject position (Lin, 2006), and the frequency-based account or its variants such as the expectation-based account (Li et al., 2010; Vasishth et al., 2013; Jäger et al., 2015). For instance, Vasishth et al. (2013) reported three self-paced reading experiments which attempted to replicate the studies conducted by Hsiao and Gibson (2003) and Gibson and Wu (2013), both of which revealed ORC advantage. Vasishth and colleagues found SRC advantage in two of these experiments, while ORC advantage was found in the last one. Based on a meta-analysis of 15 RC processing studies in Chinese, they argued that the current evidence for SRC advantage is stronger than ORC advantage in Chinese, and that this can be explained, to a certain extent, by the corpus frequency of RCs (note that the comprehensive overview given in Table 1 suggests otherwise: ORC advantage).

Certain eye-tracking (Jäger et al., 2015) and modeling (Chen et al., 2012) studies also reported SRC advantage that supports the frequency-based account or its variants. In their study combining eye-tracking and self-paced reading experiments, Jäger et al. (2015) reported SRC advantage on the embedded verb and the spillover region of the head noun. They interpreted SRC advantage to support the frequency-based account, specifically a variant of experience-based accounts called surprisal.

#### Previous studies reporting ORC advantage

Other self-paced reading studies reported shorter reading times for Chinese ORCs than SRCs, supporting the variants of the memory-based account including the storage resources metric and the integration resources metric (a.k.a. the Linear Distance Hypothesis; Chen et al., 2008; Lin and Garnsey, 2011; Gibson and Wu, 2013; Lin, 2014). For instance, Chen et al. (2008) found that SRCs took longer to read than ORCs. Critically, they observed that only low WM span readers showed this ORC advantage while high WM readers did not show any difference between SRCs and ORCs. They interpreted this finding as evidence for the memory-based account, specifically for the metric of storage resources. In another self-paced reading study, Gibson and Wu (2013) compared subject-modifying SRCs and ORCs preceded by context putatively removing temporal ambiguity, and found faster reading times on the head noun in ORCs than SRCs. They also attributed this finding to the memory-based account.

The evidence for ORC advantage in Chinese has been obtained from other methodologies, as well. In two eye-tracking studies (Sung et al., 2015, 2016), less difficulty was consistently found in reading ORCs than SRCs by means of multiple eye-movements measures especially on the head noun. Neuropsychological results also indicated that ORCs were comprehended better than SRCs in aphasic patients of agrammatism and other types (Law, 2000; Law and Leung, 2000; Su et al., 2007). Finally, Qiao et al. (2012) found ORC advantage for Mandarin Chinese by using a maze task, in which participants were required to choose one of two words presented on a frame as a possible continuation for the sentence being read. Qiao et al. (2012) showed that participants read ORCs faster than SRCs in the RC region, providing further support for ORC advantage in Mandarin Chinese from a different methodology. Moreover, previous ERP studies in Chinese also provided consistent support for ORC advantage (Yang and Perfetti, 2006; Packard et al., 2010; Yang et al., 2010). For instance, in an ERP study, Packard et al. (2010) found a larger P600 on the relativizer in subject-modifying SRCs than ORCs, and a larger P600 on the head noun in object-modifying SRCs than ORCs. The researchers interpreted these P600 effects associated with SRCs as reflecting the effect of integration cost demanded by longer filler-gap dependencies in SRCs than in ORCs. They also found a larger N400 effect on the matrix verb in subject-modifying ORCs than SRCs. They interpreted this N400 effect as indexing assignment of different thematic roles to the head noun, which is the object of the RC but the subject of the main clause, in accordance with the parallel function hypothesis (Sheldon, 1974). With these findings, the authors argued that SRCs are more difficult to process than ORCs in Chinese because of longer filler-gap dependencies in the former than in the latter.

The ERP findings in Chinese revealed that SRCs elicited a larger P600 effect than ORCs on the embedded verb (Yang and Perfetti, 2006), embedded noun (Yang et al., 2010), the relativizer and the head noun (Packard et al., 2010). Therefore, this can be interpreted as evidence for SRC processing difficulty, since P600 has usually been shown to be sensitive to syntactic processing in Indo-European languages (Hagoort, 2003) and in Chinese (Zhang and Zhang, 2008) (cf. the following section). Crucially, Packard et al. (2010) reported a P600 effect in SRCs on the head noun, which is the region where thematic argument structure is resolved and integration of filler-gap dependency takes place. For this reason, observation of a P600 on the head noun in SRCs in Packard et al. (2010) provides further support for ORC advantage in Chinese.

#### ERP components of interests

The current study examines three ERP effects generally associated with language processing: N400 and P600. The functions traditionally attributed to these components and their alternative interpretations are described below.

##### N400

N400 is a negativity between 200 and 600 ms, with a peak around 400 ms, after stimulus onset and largest over centro-parietal sites (Kutas and Federmeier, 2011). It has been shown to respond to semantic anomalies (e.g., “I take my coffee with cream and *dog*”; Kutas and Hillyard, 1980). In addition to semantic anomalies, cloze probability, world knowledge and pragmatics, figurative language and even non-linguistic contexts (e.g., congruency effects within pictures, actions, gestures and arithmetic solutions) were found to modulate the N400 effect (Kutas and Federmeier, 2011). Recently, it has been proposed that N400 indexes retrieval of lexical-semantic information (Brouwer et al., 2012, 2017).

##### P600

P600 is a positive component with a mainly posterior scalp distribution, characteristically starting at about 600 ms after the onset of the target word (Neville et al., 1991; Osterhout and Holcomb, 1992). P600 has been shown to respond to syntactic processing difficulty in a number of studies (Kaan et al., 2000; Phillips et al., 2005). It has been observed for different types of syntactic violations such as subject-verb agreement [e.g., “The spoiled child *throw* the toys on the floor.” (instead of the grammatically inflected verb *throws*), Hagoort et al., 1993] and for garden-path sentences [e.g., “The broker persuaded to sell the stock was sent to jail.” (Osterhout and Holcomb, 1992)]. It is thought that P600 reflects syntactic reanalysis processes in such sentences. P600 was also reported in sentences containing no violation or garden-path, but in which syntactic integration was difficult due to long-distance dependency (e.g., “Emily wondered who the performer in the concert had imitated for the audience's amusement.” Kaan et al., 2000), where the object (*who*) must be attached to its verb (*imitated*). Recent studies challenged the claimed division of labor between N400 and P600, indexing semantic and syntactic processing respectively, a claim deeply engrained in the literature (Brouwer et al., 2017). For instance, semantically anomalous words in Dutch sentences failed to elicit an N400 effect, but generated a P600 effect compared to semantically congruous words (Hoeks et al., 2004). Attempts were made to reconcile these “semantic illusions” within the scope of neurocognitive models. For instance, the retrieval-integration account claims that N400 indexes retrieval of lexical-semantic information, on the premises that retrieval of information pertaining to a word that is activated by prior context is easier than a word not so activated (Brouwer et al., 2012, 2017). P600, on the other hand, reflects integration of the retrieved word into the representation of the utterance. Regardless of whether P600 indexes construction and deconstruction of syntactic relations (Gouvea et al., 2010), or difficulties encountered in constructing a mental representation (Brouwer et al., 2012), it is still commonly regarded as a difficulty with the construction that is being integrated with the prior context.

#### Aims and predictions of the current study

As outlined in Table 1, there were conflicting findings in research into Chinese RC processing and lack of consensus about which RC processing account provides satisfactory explanation. A previous self-paced reading study suggested that WM affects the reading times of SRCs and ORCs (Chen et al., 2008). As the possible effect of verbal WM capacity on processing patterns of Chinese RCs was not adequately addressed in previous studies and was not investigated by using ERPs at all, the present ERP study was conducted to record participants' brain waves when they read Chinese sentences with RCs. The participants' WM capacity was measured by a forward digit span test and an operation span test to determine the relationship between this factor and the magnitude of the difference between processing SRCs and ORCs. To that end, we divided the participants into two groups: the high WM group and the low WM group.

The memory-based account predicts that an SRC disadvantage holds for processing Chinese RCs. The integration resources metric, or the Linear Distance Hypothesis, of this account would predict a P600 effect elicited by SRCs on the head noun, reflecting difficulty of integrating a more distant filler-gap dependency.

In contrast with the memory-based account, the Structural Distance Hypothesis and the frequency-based account would predict ORC disadvantage. The Structural Distance Hypothesis does not make a clear prediction about where the ORC disadvantage should be observed (O'Grady et al., 2003); however, it is reasonable to assume that this processing asymmetry should be observed after the RC reading is unambiguously signaled; i.e., on the relativizer and the head noun. Likewise, the frequency-based account (including the variants such as expectation/experience-based approaches) does not clarify exactly where this disadvantage should be observed (Qiao et al., 2012), as it depends on multiple factors such as potential ambiguities in parsing and the point where the parser predicts an RC (Jäger et al., 2015). As can be inferred from Table 1, the majority of reaction-time studies conducted on Chinese RCs and supporting the frequency-based account and the Structural Distance Hypothesis reported greater reaction times for ORCs on the head noun in general, and occasionally on the embedded verb and noun, and less frequently on the relativizer; hence it is predicted that a P600 should be elicited by SRCs on the head noun in particular.

In terms of the WM span, the Structural Distance Hypothesis and the frequency-based account do not predict a difference between the low and high WM groups in the P600 effect that may be observed in ORCs. This is because the structural and frequency/expectation/experience-related information is independent of having a higher or lower WM span, and, instead, dependent on statistical patterns of particular languages. The memory-based account, on the other hand, assumes that linguistic integration and storage processes access the same pool of WM resources (Gibson, 1998). However, this account is agnostic as to whether the particular computational resources recruited in sentence processing tap into the general pool of memory resources, consistent with the shared resource/domain-general view of WM (Just and Carpenter, 1992), or whether they constitute a modular linguistic memory pool, consistent with the dedicated resource/domain-specific view of WM (Waters and Caplan, 1996). The dedicated resource view argues that the WM component utilized in sentence comprehension is separate from the component measured in standard tests of WM (Caplan and Waters, 1999). The verbal WM system is conceptualized as comprising two distinct subsystems. One of these subsystems is utilized in sentence-level interpretive processes, which consist of assigning syntactic structure to sentences and using that structure to comprehend the sentence. The other subsystem is engaged in post-interpretive processing, which refers to the use of sentence meaning to perform other tasks such as reasoning and planning actions. Hence, on the basis of studies on normal participants and patients with poor short-term memory and patients with aphasia, Caplan and Waters (1999, 2013) postulate a specialization in the verbal WM system for processing syntactic structure of sentences. The shared resource view, on the other hand, postulates a single, constrained capacity and its dynamic allocation by the central executive to cognitive tasks (Just and Carpenter, 1992). According to this theory, individual differences in capacity can explain systematic differences in performance within a given task domain, such as language. Hence, under conditions with high task demands (e.g., syntactic ambiguity or integration), individuals with a higher WM capacity should be capable of computing or storing information better than those with a lower WM capacity.

Thus, the shared resource view would predict that the P600 effects for SRCs should be different for the high and low WM span readers, as the former group has greater WM resources at their disposal, hence the longer filler-gap dependency in SRCs would not cause as great a cost to their capacity as that of the low WM span group. The dedicated resource view, on the other hand, would not predict any difference between the two WM groups in terms of the P600 effects in SRCs, as the linguistic long-distance dependency should tap into the dedicated linguistic memory pool (e.g., syntactic WM; Fiebach et al., 2001).

## Methods

This study was carried out in accordance with the recommendations of the Social and Behavioral Research Ethical Principles and Regulations of National Taiwan University with written informed consent from all subjects. All subjects gave written informed consent in accordance with the Declaration of Helsinki. The protocol was approved by the Research Ethics Committee of National Taiwan University.

### Participants

Twenty-four right-handed, healthy graduate and undergraduate students (11 females; Age Mean = 23, *SD* = 2.5) from National Central University took part in the experiment. All of them were native speakers of Mandarin Chinese and had normal or corrected-to-normal vision and hearing. They were all right-handed. They received monetary compensation for participating in the experiment.

### Materials

One hundred and twenty pairs of experimental sentences with object-modifying subject relative clauses (*N* = 120) and object relative clauses (*N* = 120) were created as shown in Table 2. All experimental items consisted of 7 words (each consisting of 1–4 characters), corresponding to 7 frames of presentation. All experimental items had two animate NPs, one as the head of the RC and the other as the object or the subject of the RC. Because the two animate NPs were equally likely to be the head of the RC and the subject/object of the RC; i.e., they were semantically reversible, syntactic processing was necessary to map thematic roles to the arguments. Importantly, the conditions were minimally different from each other. Exactly the same words were used across the conditions, and only the order of the embedded nouns and verbs (e.g., *star* and *beat* in Table 2) was changed to manipulate the RC type.

**Table 2:** Example experimental stimuli.

**Sentence type**		**Embedded N/V**	**Relativizer**	**Head noun**
Subject relative clause	民眾討厭那個	毆打明星	的	攝影師
	people hate the	beat star	*de*	photographer
	People hate the photographer who beat the star			
Object relative clause	民眾討厭那個	明星毆打	的	攝影師
	people hate the	star beat	*de*	photographer
	People hate the photographer who the star beat			

As exemplified in Table 2, each sentence with a relative clause contained a demonstrative + classifier compound, which corresponds to *this* (這個) or *that* (那個) in English. Previous literature has suggested that such demonstrative and classifier compounds are frequently used in Chinese relative clauses, and may aid comprehension of these structures by signaling their presence in advance of encountering a relative clause later in the sentence (Wu et al., 2010; Wu, 2011; Tai and Du, 2016).

One hundred and eighty filler sentences were also created. Among these fillers, 120 sentences were prepared by using the same nouns and verbs as in the experimental conditions in a way that they did not form an RC due to omission of the relativizer. These 120 fillers aimed to reduce the participants' expectation of an RC continuation and to encourage them not to focus on the dichotomy between SRCs and ORCs. The remaining 60 fillers had various structures with their length ranging from 3 to 8 words.

### Procedure

The experimental items (and the fillers with the same nouns and verbs as the experimental items) were randomized and distributed to three lists. The items were counterbalanced across the three lists such that an equal number of each condition (*N* = 40 per condition) appeared in each list and no participant saw more than one version of each item.

Each sentence in the experiment was paired with a comprehension question. For the comprehension questions about the experimental sentences, one-third of them required the readers to understand the syntactic/semantic relations between the main/embedded NP and the matrix verb, while two-thirds of them involved the relations between the main/embedded NP and the verb in the embedded clause. Comprehension questions about the fillers probed general understanding of the sentences.

Before the experiment commenced, participants were fitted with an elastic electrode cap and seated in a quiet room at a viewing distance of 80 cm from the monitor. Each Chinese character constituting the visual stimuli subtended a visual angle of approximately 1° vertically and was presented in DFKai-SB font in white against black background. Each trial was initiated with a fixation cross presented for 500 ms. The materials were presented at the center of computer screen word by word with a 300-ms presentation time and 400-ms inter-stimulus interval, summing up to 700 ms of stimulus-onset asynchrony.

Participants were asked to read the words for comprehension and they answered a true-false comprehension question at the end of each sentence by pressing one of the two buttons indicated on the keyboard. They received immediate feedback for each answer. Participants were asked to try not to blink starting from the fixation cross until the presentation of the question. There were four sessions, each of which lasted about 10 min. Participants determined their own pace between trials, pausing any time they wanted after each trial or between sessions. In total, the ERP experiment took approximately 1 h to complete.

### EEG recording and preprocessing

Electroencephalography (EEG) was continuously recorded during the experiment from 64 Ag/AgCl electrodes, 62 of which were embedded in an elastic cap (Quick-Cap, Neuromedical Supplies, Sterling, TX). The remaining two electrodes were placed on the right and left mastoids. All channels were referenced to a channel located between Cz and CPz, and were re-referenced offline to the average of the two mastoids. A ground electrode was placed on the forehead anterior to the Fz electrode. Vertical and horizontal electrooculograms (EOG) were recorded bipolarly from electrodes placed above and below the right eye and on the outer canthi of each eye, respectively. Data was sampled at 250 Hz and digitized with 24-bit resolution. All channels were amplified by SYNAMPS2 (Neuroscan, Inc., El Paso, TX) with a band-pass of 0.05–70 Hz. Interelectrode impedance was kept below 5 kΩ. We initially analyzed the ERPs on the relativizer and the head noun in separate 700-ms time windows with a 100-ms prestimulus baseline immediately preceding each one of the target words. However, it has been suggested to us by the editor of the current article that because part of the P600 effect elicited by ORCs on the relativizer corresponded to this baseline period adopted for the head noun, this not only led to cutting off the ongoing P600 effect on the relativizer, but also introduced a confound in the ERPs on the head noun, such that the P600 effect elicited by SRCs on the head noun was exaggerated (see Steinhauer and Drury, 2012 for a discussion of similar baseline problems in ERP studies). In other words, as the ERPs for ORCs were more positive than those for SRCs during the relevant baseline, ORC waveform on the head noun was artificially shifted toward the negative polarity, creating at least part of the positivity for SRCs on the head noun. The results of this analysis on the head noun with the potentially problematic baseline are given in the Supplementary Material. For these reasons, ERPs reported below were computed for a single, multiword epoch of 1,500 ms comprising the relativizer and the head noun with a 200-ms prestimulus baseline preceding the onset of the relativizer, where the main effect of RC-type or any one of its interactions was not significant (*p*s ≥ 0.135). Linear regression was used to estimate and correct the contribution of blink artifacts to the EEG (Semlitsch et al., 1986). Trials containing horizontal eye movement, nonblink vertical eye movement, A/D saturation, or with a baseline drift exceeding 70 microvolts in any channel were rejected.

### Statistical analyses

Two separate global ANOVAs were conducted on ERPs for the medial electrodes and the lateral electrodes. The medial electrodes consisted of Fz, Cz, and Pz for the analysis of the N400 and P600 time windows on the relativizer and the N400 time window on the head noun, and Fz, Cz, and POz for the analysis of the P600 time window on the head noun. The lateral electrodes consisted of the pairs of F7-F8, F3-F4, C5-C6, P3-P4, and T7-T8 for the analysis of the N400 and P600 time windows on the relativizer and the N400 time window on the head noun region, and F7-F8, F3-F4, C5-C6, PO3-PO4, and T7-T8 for the analysis of the P600 time window on the head noun. PO electrodes were used for the analysis of P600 time window on the head noun because the P600 effect in this region had a posterior-occipital distribution that was more robust in POz, PO3, and PO4 electrodes than Pz, P3, and P4. C5-C6 electrode pairs were used in the lateral analyses instead of C3-C4 because C3 malfunctioned in some of the participants. Each ANOVA had two within-subject factors: RC-type (SRC vs. ORC) and electrodes (three electrodes for medial ANOVA and five pairs for lateral ANOVA) and one between-subjects factor (WM group). The lateral ANOVA had an additional within-subjects factor of hemisphere (left vs. right). If a global ANOVA yielded a significant (*p* < 0.05) interaction including the factor RC-type, it was followed up with step-down ANOVAs to elucidate the nature of the interaction. Greenhouse-Geisser correction for deviations from sphericity in the data was applied wherever applicable. The following time windows were selected on the basis of previous research and 50-ms moving window analyses of the ERPs: N400 on the relativizer & head noun: 300–500 ms; P600 on the relativizer: 600–1,000 ms; P600 on the head noun: 450–800 ms.

### Operation span

An automated version of the operation span test (Unsworth et al., 2005), which is based on the operation span (Ospan) test designed by Turner and Engle (1989), was used to obtain a measure of the participants' WM capacity. The Ospan test required the participants to judge accuracy of mathematical equations presented on the computer screen (e.g., (3 × 4) + 1 = 16?), while trying to remember a set of letters displayed on the screen. The size of the letter sets ranged from 3 to 7, randomly ordered for each participant. The Ospan test consisted of two sessions. In the first session, the participants judged accuracy of mathematical equations only. This not only served as a practice session, but it also allowed the program to calculate the participants' individual speed of mathematical calculation. This time (plus 2.5 SD) was used in the second session to present the equations. In the second session, from which the Ospan scores were obtained, the participants were required to remember the sets of letters presented between equations and to enter the letters in the correct order by using the keyboard when prompted by dashes on the screen. The participants received feedback for accuracy in judging equations and remembering letters. A threshold of 85% accuracy on math equations was imposed for all the participants in order to engage them in both math equations and letter recall. Ospan scores were calculated on the basis of the perfectly recalled sets. Six participants failed to pass the accuracy threshold of 85%, while 18 participants successfully completed the procedure. The Ospan scores across the participants passing the accuracy threshold ranged from 0.59 to 0.97 (Mean = 0.84; *SD* = 0.10).

### Forward digit span

A forward digit span test was used to obtain another measure of WM. For this test, the participants were seated in front of a computer screen and asked to try to remember the order of the digits visually presented on the center of the screen one at a time and, when they saw the recall cue on the screen, to write down the digits on an answer sheet in the order they were presented. While viewing the digits, the participants were asked not to take notes, speak the digits aloud, do any gestures or move any part of their body to help them in the process. The digits had 10 levels (ranging from 4-digit numbers to 13-digit numbers), each comprising 5 trials. Level 4 was used as practice items. At the end of the test, each participant's forward digit span score was found in this manner: Answers were checked starting from the lowest level (level 5) and marked as correct if they correctly indicated the correct digits in the correct sequence. If the participant made three or more errors in one level, marking was terminated and the participant was given the score of the preceding level. The digit span scores across the participants ranged from 6 to 12 (Mean = 9.33; *SD* = 1.55).

A two-tailed Pearson correlation was conducted between the two WM tests implemented in the present study. A positive correlation was observed between the forward digit span and operation span scores (*r* = 0.700, *N* = 18, *p* = 0.001). Because there was a highly significant correlation between the two WM measures and because Ospan scores could not be elicited from six participants due to accuracy limitation, the digit span scores were considered in dividing participants into high (digit span at or above the median 10, *N* = 13, Age Mean = 22) and low (digit span below the median 10, *N* = 11, Age Mean = 23) WM groups. The groups did not differ in terms of age [*t*_(12.075)_ = 1.019, *p* = 0.328]. The Ospan and digit span scores were proportionally similar within the WM groups [Low WM Group: Ospan (*M* = 0.78; *SD* = 0.12) & Digit Span (*M* = 7.9; *SD* = 0.83); High WM Group: Ospan (*M* = 0.90; *SD* = 0.04) & Digit Span (*M* = 10.54; *SD* = 0.78)]. Instead of treating WM as a continuous variable, we chose to divide the participants into high and low WM groups. This was because the current study was partly motivated by Chen et al. (2008), who found that WM modulated reading times for SRCs and ORCs and who, similarly, divided their participants into high and low WM groups. However, we still conducted our statistical analyses by treating the WM scores as a continuous variable where necessary, as reported below.

## Results

### Comprehension results

The accuracy and reaction times (of accurate responses only; after removal of outliers beyond 2 SD from the mean) of the participants' responses to comprehension questions are given in Table 3 for the high and low WM groups. The comprehension accuracy and reaction times were subjected to two separate repeated-measures ANOVAs with RC types (SRC & ORC) as the within-subjects factor and WM (high & low) as the between-subjects factor.

**Table 3:** Mean accuracy and reaction times on comprehension questions.

	**Working memory capacity**							
**Sentence Type**	**Low**				**High**			
	**Accuracy**		**Reaction times**		**Accuracy**		**Reaction times**	
	**Mean (%)**	**SD (%)**	**Mean (ms)**	**SD (ms)**	**Mean (%)**	**SD (%)**	**Mean (ms)**	**SD (ms)**
SRC	74	15	1,482	386	83	8	1,229	441
ORC	76	15	1,347	222	84	8	1,235	406
Filler	91	8	1,346	233	96	3	1,195	287

Fillers, which were not included in statistical analysis, were answered with a mean accuracy of 94%, followed by ORCs (80%) and SRCs (78%). There was no significant difference between SRCs and ORCs [*F*_(1, 22)_ = 0.36]. The between-subjects factor of WM showed significant difference [*F*_(1, 22)_ = 4.95, *p* = 0.037] due to the fact that the high WM group answered the comprehension questions in general more accurately (83%) than the low WM group (75%). There was no interaction between RC type and WM [*F*_(1, 22)_ = 0.02]. Reaction times for the comprehension questions did not reveal a significant effect of RC type [*F*_(1, 22)_ = 1.27] or WM [*F*_(1, 22)_ = 1.55]. There was no interaction between RC type and WM [*F*_(1, 22)_ = 1.95], either, although the low WM group took numerically longer to answer comprehension questions for SRCs than ORCs (*p* = 0.087, SE = 0.075). In summary, the behavioral data did not show any significant differences between SRCs and ORCs, nor an interaction between RC type and WM.

### ERP results

**Figure 1:**
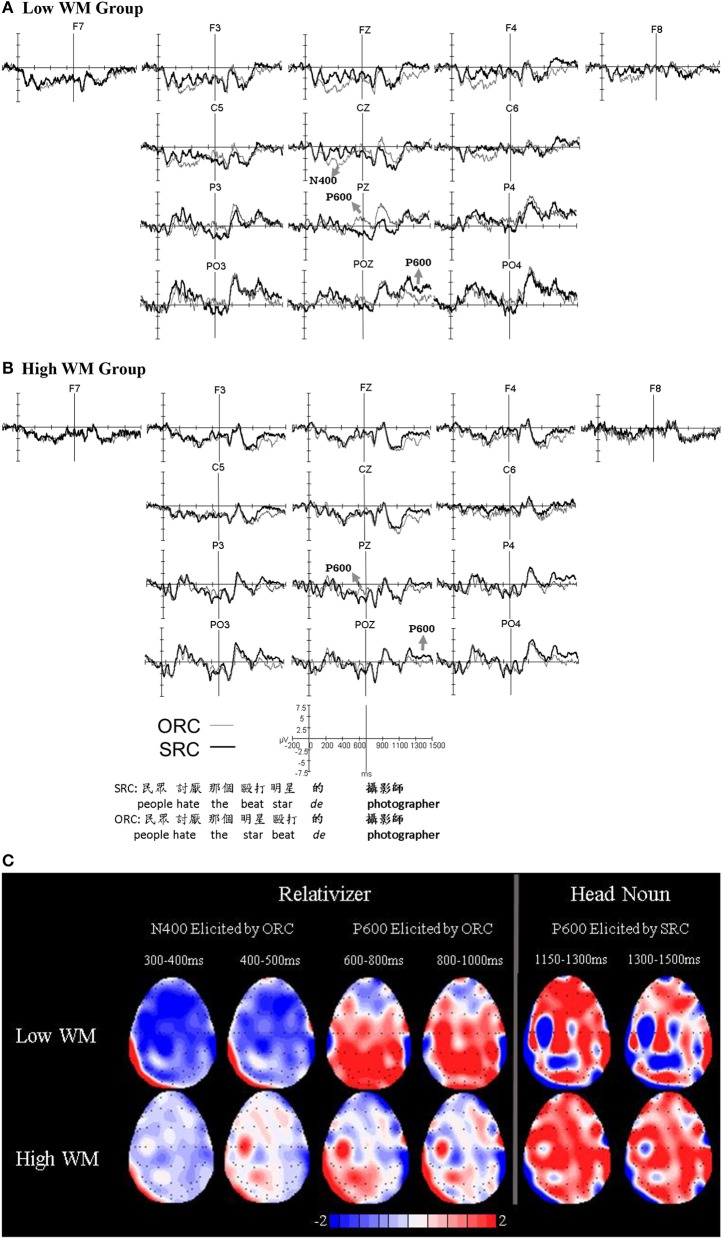
Grand average ERPs on the relativizer and the head noun for the low working memory group **(A)** and the high working memory group **(B)**. Topographic distributions of the difference waves showing the ERP effects elicited by ORC and SRC on the relativizer and the head noun for the low and high working memory groups **(C)**.

At the artifact rejection step, four participants were removed from the analysis due to excessive loss of trials. The remaining participants contributed an average of 67.5% of the trials to the analysis (number of accepted trials: SRC, Mean = 27, Range = 13–38; ORC, Mean = 27, Range = 13–38). The number of rejected trials did not differ statistically across conditions [*t*_(19)_ = 0.189, *p* = 0.852]. After exclusion of these participants, there remained unequal numbers of participants in the two WM groups. Therefore, the Ospan scores were used to assign two participants who had the median digit span score but a relatively low operation span score to the low working memory group to reach an equal number of participants in the low WM group (*N* = 10) and the high WM group (*N* = 10) for ERP analyses. The Ospan and digit span scores were proportionally similar within the WM groups [Low WM Group: Ospan (Mean = 0.82; *SD* = 0.11) & Digit Span (Mean = 8.4; *SD* = 1.11); High WM Group: Ospan (Mean = 0.92; *SD* = 0.03) & Digit Span (Mean = 10.70; *SD* = 0.78)]. The multiword region was divided into the Relativizer and Head Noun regions as shown in Figure 1. Table 4 summarizes the ANOVA *F*-values for the main effect of RC-Type and its interactions with the other factors on the Relativizer and Head Noun regions. The main effect of RC-type or any one of its interactions was not significant in the 200-ms prestimulus baseline (*p*s ≥ 0.135). We also analyzed the relative clause verb and noun as a multiword region; however, the results obtained were confounded by lexical differences (verb vs. noun), hence they are not included here, but presented in the Supplementary Material.

**Table 4:** Summary of ANOVAs for the Relativizer and the Head Noun Regions.

		**Relativizer**		**Head noun**	
**Source**	***df***	***f*****-Values**			
		**N400**	**P600**	**N400**	**P600**
**MEDIAL ANOVA**		**300–500 ms**	**600–1,000 ms**	**1,000–1,200 ms**	**1,150–1,500 ms**
**RC-Type**	**1,18**	7.652^*^	**1.139**	**0.579**	4.626^*^
**RC-Type × Electrodes**	**2,36**	**3.371**	9.004^**^	**2.535**	**1.052**
**RC-Type × WM**	**1,17**	5.316^*^	**0.758**	**0.419**	**0.019**
**RC-Type × Electrodes × WM**	**2,34**	**0.988**	**2.224**	**1.745**	**0.647**
**LATERAL ANOVA**					
**RC-Type**	**1,18**	11.349^**^	**0.194**	**0.856**	**2.662**
**RC-Type × Electrodes**	**4,72**	**0.608**	**2.035**	**1.038**	**2.097**
**RC-Type × Hemisphere**	**1,18**	**2.389**	**0.654**	**0.020**	**0.028**
**RC-Type × Hemisphere × Electrodes**	**4,72**	**0.836**	**0.841**	**0.674**	**0.696**
**RC-Type × WM**	**1,17**	**2.768**	**0.921**	**0.374**	**0.126**
**RC-Type × Electrodes × WM**	**4,70**	**0.749**	**0.551**	**0.303**	**0.203**
**RC-Type × Hemisphere × WM**	**1,16**	**0.184**	**0.012**	**0.009**	**0.074**
**RC-Type × Hemisphere × Electrodes × WM**	**4,68**	**1.623**	**0.503**	**0.164**	**0.143**

* *p < 0.05*.

** *p < 0.01*.

#### Relativizer

As shown in Figure 1, ORCs were associated with more negative-going signals than SRCs, which was evidenced by the main effect of RC-Type between 300 and 500 ms in both the medial [*F*_(1, 18)_ = 7.652, *p* = 0.013, η^2^ = 0.298] and the lateral ANOVA [*F*_(1, 18)_ = 11.349, *p* = 0.003, η^2^ = 0.387].

Importantly, the ERPs elicited by SRCs and ORCs showed differences between the WM groups as revealed by the significant interaction of RC-Type^*^WM in the same time window in the medial ANOVA [*F*_(1, 17)_ = 5.316, *p* = 0.033, η^2^ = 0.228]. Step-down analyses showed that this interaction was due to the fact that ORCs elicited more negative ERPs than SRCs between 300 and 500 ms only for the low WM group [*F*_(1, 9)_ = 9.704, *p* = 0.012, η^2^ = 0.519], whereas there was no significant difference between the two RC-types for the high WM group [*F*_(1, 9)_ = 0.157, *p* = 0.701, η^2^ = 0.017]. This difference between the two WM groups can be explained by the presence of a broadly-distributed N400 in the low WM group elicited by ORCs between 300 and 500 ms, as illustrated by the topographic distribution in Figure 1C.

Finally, a significant interaction between RC-Type and Electrodes was observed between 600 and 1,000 ms in the medial ANOVA [*F*_(2, 36)_ = 9.004, *p* = 0.003, η^2^ = 0.333]. Step-down analyses revealed that this interaction was due to the presence of a positive-going waveform elicited by ORCs compared to SRCs between 600 and 1,000 ms at posterior electrodes, which reached significance at Pz [*F*_(1, 19)_ = 6.602, *p* = 0.019, η^2^ = 0.258], but not at the other medial electrodes [*Fs*_(1, 19)_ ≤ 0.930, *ps* ≥ 0.347]. According to its time frame and scalp distribution, this mid-posterior positivity is interpreted as a P600 effect. The interaction of RC-Type^*^Electrodes^*^WM did not reach significance in the medial ANOVA [*F*_(2, 34)_ = 2.224, *p* = 0.143, η^2^ = 0.110] within the P600 time frame (600–1,000 ms), which means that the P600 effect was observed for both WM groups. However, as can be observed in the topographic distribution in Figure 1C, the P600 effect persisted longer in the low WM group than the high WM group, while it was reduced especially after 700 ms in the high WM group. Indeed, analyses with shorter time windows revealed that the interaction of RC-Type^*^Electrodes^*^WM approached significance between 750 and 900 ms in the medial analysis [*F*_(2, 34)_ = 3.125, *p* = 0.077, η^2^ = 0.148], and reached significance between 850 and 900 ms [*F*_(2, 34)_ = 4.711, *p* = 0.022, η^2^ = 0.207]. Step-down ANOVAs showed that this interaction was due to the presence of significantly more positive-going ERPs elicited by ORCs than SRCs between 850 and 900 ms only in the low WM group at Pz [*F*_(1, 9)_ = 5.144, *p* = 0.050, η^2^ = 0.364], but not at other electrodes [*Fs*_(1, 9)_ ≤ 0.771, *ps* ≥ 0.403]. However, there was no significant difference between SRCs and ORCs in the same time window for the high WM group at any one of the medial electrodes [*Fs*_(1, 9)_ ≤ 0.774, *ps* ≥ 0.402].

In order to examine whether treating WM scores as a continuous variable would increase the magnitude of this interaction between RC-Type, Electrodes and WM, which might have been reduced due to the median-split procedure adopted, a correlation analysis was performed by treating the WM scores (both digit span and operation span scores) as continuous variables. Specifically, the difference ERPs (ERPs elicited by ORCs minus ERPs elicited by SRCs) at Pz, where the P600 effect was the strongest, in the P600 time window (600–1,000 ms) were entered into correlation analyses with the WM scores. These analyses yielded a significant correlation between the P600 difference ERPs and the operation span scores (*r* = −0.574, *n* = 13, *p* = 0.040), but not between the P600 difference ERPs and the digit span scores (*r* = −0.178, *n* = 20, *p* = 0.454). This correlation analysis showed that the higher the participants' operation span scores, the more reduced the P600 effect.

In summary, an N400-P600 complex was observed on the relativizer that was modulated by WM span, such that these effects were more pronounced for the low WM group. These results are not predicted by the memory-based account; nonetheless, it does not contradict with this account, either. Although the results seem to support the frequency-based account and the Structural Distance Hypothesis by showing an ORC disadvantage, these accounts fail to explain the modulatory role of individual WM span. Instead, it is more plausible to consider this N400-P600 complex as reflecting detection and reanalysis of syntactic ambiguity on the relativizer in ORCs, as discussed below.

#### Head noun

The ERPs on the head noun, as shown in Figure 1, did not reveal any significant difference between the conditions in the N400 time window (1,000–1,200 ms in the multiword time window, which corresponds to 300–500 ms following the onset of the head noun; *F*s ≤ 2.535, *p*s ≥ 0.104).

As shown in Figure 1, there was a broadly-distributed positivity elicited by SRCs compared to ORCs starting at approximately 1,150 ms and lasting until the end of the analysis window (1,500 ms). Indeed, this observation was supported by the significant main effect of RC-Type between 1,150 and 1,500 ms in the multiword time window (corresponding to 450–800 ms following the onset of the presentation of the head noun) in the medial analysis [*F*_(1, 18)_ = 4.626, *p* = 0.045, η^2^ = 0.204] due to more positivity elicited by SRCs than ORCs. No significant main effect of RC-Type was found in the lateral analysis [*F*_(1, 18)_ = 2.662, *p* = 0.120, η^2^ = 0.129]. Although the interaction between RC-Type and Electrodes did not reach significance within this time window in the lateral [*F*_(4, 72)_ = 2.097, *p* = 0.110, η^2^ = 0.104] or medial [*F*_(2, 36)_ = 1.052, *p* = 0.343, η^2^ = 0.055] ANOVAs, it was significant for a shorter time window between 1,300 and 1,500 ms in the multiword time window (corresponding to 600–800 ms following the onset of the head noun) in the lateral ANOVA [*F*_(4, 72)_ = 2.989, *p* = 0.039, η^2^ = 0.142], but not in the medial ANOVA [*F*_(2, 36)_ = 1.647, *p* = 0.214, η^2^ = 0.084]. The step-down analyses showed that this interaction of RC-Type^*^Electrodes in the lateral analysis was due to a more positive-going waveform associated with SRCs than those associated with ORCs at the PO3-PO4 electrode pair in the P600 time window [*F*_(1, 19)_ = 5.935, *p* = 0.025, η^2^ = 0.238], but not in any other electrode pairs [*F*s_(1, 19)_ ≤ 1.605, *p*s ≥ 0.221]. Overall, although this positivity elicited by SRCs compared to ORCs on the head noun was broadly-distributed with a lateral posterior-occipital component, but without a midline posterior peak that is characteristic of the P600 effect, we tentatively interpret it as an atypically distributed P600 effect due to its time window and discuss it as such in the following sections.

As can be seen in Table 4, the between-subjects factor of WM did not affect the P600 effect elicited by SRCs on the head noun. Furthermore, none of the 50-ms moving-window analyses conducted in the P600 time window (1,150–1,500 ms in the multiword time window) revealed any significant interaction of RC-Type^*^WM or RC-Type^*^Electrodes^*^WM in the medial or lateral analyses (*F*s ≤ 0.951, *p*s ≥ 0.370). In order to examine whether treating WM scores as a continuous variable would reveal any significant effects that might have been overlooked by the median-split procedure adopted, a correlation analysis was performed by treating the WM scores (both digit span and operation span scores) as continuous variables. Specifically, the difference ERPs (ERPs elicited by SRCs minus ERPs elicited by ORCs) at the three medial electrodes (Fz, Cz, POz) in the P600 time window (1,150–1,500 ms in the multiword time window), and at the PO3-PO4 electrode pair in the shorter time window (1,300–1,500 ms in the multiword time window) were entered into correlation analyses with the digit and operation span scores. These analyses did not yield any significant correlation, either (*p*s ≥ 0.096).

Although somewhat atypically distributed, the P600 effect elicited by SRCs on the head noun might indicate syntactic integration cost associated with Chinese SRCs, providing tentative support for the integration resources metric of the memory-based account, since the head noun is the site where filler-gap integration takes place.

## Discussion

The aim of the present study was to investigate the processing patterns of subject and object relative clauses in Mandarin Chinese by using ERPs and to examine any effects of WM on these processing patterns. It was found that ORCs were associated with an N400-P600 complex on the relativizer, which was more robust for the low WM group. ERPs on the head noun, on the other hand, revealed a broadly-distributed P600 effect elicited by SRCs. Overall, the current results are in parallel with the inconsistent findings reported in the literature on Chinese relative clause processing. As summarized in Table 5, the current findings seem most compatible with the integration metric of the memory-based account (Linear Distance Hypothesis), although they do not necessarily rule out the frequency-based account or the Structural Distance Hypothesis.

**Table 5:** Comparison of the current findings with predictions of the sentence processing accounts.

	**Observed effects**		**Predictions of linear distance hypothesis**		**Predictions of structural distance hypothesis**	
	**SRC**	**ORC**	**SRC**	**ORC**	**SRC**	**ORC**
Relativizer	—	N400 & P600 (for LWM)	—	—	—	P600
Head noun	P600 (for both WM Groups)	—	P600^*^	—		

* *The Linear Distance Hypothesis does not make a prediction about whether there should be an effect of WM group or not*.

The presence of N400-P600 complex for ORCs on the relativizer might be due to a syntactic reanalysis or ambiguity resolution. When readers encountered the embedded verb in ORCs, they might have postulated two potential continuations for the sentence: an RC reading which predicts the next word to be a relativizer, and a subordinate clause reading (such as a that-clause) which predicts the next word to be a noun serving as the object of the subordinate clause. This ambiguity is then resolved on the following word, which is the relativizer. The presence of the relativizer unambiguously signals an RC reading, hence leading to a disambiguation and/or reanalysis. In SRCs, on the other hand, the presence of an RC is unambiguously signaled earlier when the embedded RC verb is encountered. This interpretation is consistent with previous research that has shown that N400 is highly correlated with expectancy and predictability (i.e., cloze probability) of a given word (Kutas and Federmeier, 2011; Lee et al., 2012). In the present study, the participants may have anticipated the upcoming relativizer in SRCs, but not in ORCs, resulting in an N400 effect for the latter. Due to the fact that the anticipation of a relativizer influenced the parsing choices (a relative clause parse or a subordinate clause parse), the participants may have needed to reanalyze the current parse when they reached the relativizer in ORCs, leading to the observed P600. This interpretation is also consistent with previous studies that associated the P600 effect with reanalysis of syntactic ambiguities (Osterhout and Holcomb, 1992). Therefore, the present finding of an N400-P600 complex on the relativizer might have arisen from unexpected continuation in ORCs, and reanalysis of the resulting syntactic ambiguity.

In the present study, the N400-P600 complex associated with the relativizer in ORCs was observed in the low WM group with much greater magnitude than in the high WM group. Given that N400 has been associated with the cloze probability of incoming words as discussed in the preceding paragraph, the reduced and less obvious N400 pattern for the high WM group indicates that they entertained the possibility of a relative clause parse, hence the upcoming relativizer, and postulated both potential readings of the sentence. Furthermore, this advantage seems to have enabled them to reach the correct parse of the structure without initiating a substantial reanalysis. This interpretation is consistent with previous studies which showed that individuals with a high WM span comprehend garden-path sentences (which required reanalysis) better than those with a low WM span (e.g., Daneman and Carpenter, 1983) and supports the shared resource view of WM resources (Just and Carpenter, 1992). Furthermore, it has been shown that high WM capacity allows these individuals to maintain multiple interpretations of a particular structure (Just and Carpenter, 1992).

A positivity with broad distribution was elicited by SRCs for both WM groups on the head noun, which is the NP modified by the RC. Although its scalp distribution was somewhat atypical (without a maximum at posterior midline that is characteristic of a conventional P600 effect), it was interpreted as a P600 effect due to its time window (450–800 ms after the onset of the head noun). In addition to syntactic reanalysis and repair, P600 was also reported for sentences containing no violation or garden-path, but in which syntactic integration was difficult due to long-distance dependency (Kaan et al., 2000; Phillips et al., 2005). A similar finding for Mandarin Chinese was reported by Packard et al. (2010), who also found a P600 effect on the head noun for object-modifying SRCs. In line with their interpretation, this P600 effect might be indicative of syntactic integration cost associated with Chinese SRCs. Some of the previous self-paced reading studies also revealed similar results (Gibson and Wu, 2013); namely, ORCs were read faster on the head noun than SRCs. More recent eye-tracking studies also showed a similar pattern (Sung et al., 2015, 2016), reporting shorter gaze durations and less regressions on the head noun for ORCs than for SRCs.

The current findings provide partial support for the Linear Distance Hypothesis, which is conceptualized in terms of the integration resources metric of Gibson's Dependency Locality Theory (DLT; Gibson, 1998, 2000). As introduced earlier, DLT utilizes two metrics to account for sentence processing dynamics, namely, storage resources and integration resources. While storage resources are based on incomplete head-dependencies that are predicted on each sentence increment, integration resources refer to the process of establishing connection between the incoming word and the current syntactic structure (Gibson, 1998, 2000). In Chinese, integration cost, calculated by the distance of the dependency (linear distance between dependents; i.e., filler and gap), is greater for SRCs than ORCs due to longer distance between the head noun (i.e., the filler) and its location of extraction (i.e., the gap). The metric of integration resources predicts processing costs for SRCs on the head noun, which is the site where the filler-gap dependency is resolved and the head noun is attached to its RC verb. Although the P600 elicited by SRCs on the head noun offers some support for the Linear Distance Hypothesis, this account cannot explain the N400 and P600 effects associated with ORCs on the relativizer.

The fact that there was no modulatory effect of WM, measured by the digit span or the operational span, on the P600 elicited by SRCs on the head noun suggests that both WM groups exhibited a syntactic integration cost on the head noun regardless of their WM capacity. Furthermore, the correlation analysis, in which WM was treated as a continuous variable, also failed to reveal any significant correlation between the scores of WM span from both tests and the amplitude of P600 on the head noun. No previous ERP study tested the effect of WM on RC processing patterns in Chinese. Only a self-paced reading study was conducted in which WM was shown to affect RC processing in Mandarin Chinese (Chen et al., 2008), in that SRCs took longer to read than ORCs only for the low WM group. These findings suggest that having a higher WM span provides a processing advantage. This advantage may reveal itself as an ability to better predict upcoming words, as reflected in the reduced N400-P600 complex on the relativizer for the high WM group in the current study. However, the lack of evidence for any WM effect on the head noun suggests that having a high WM span did not cancel the cost of integrating a long-distance dependency on the head noun. Therefore, it seems that the processing asymmetry between Chinese SRCs and ORCs exists regardless of any processing advantage afforded by having a high WM span, supporting the dedicated resource/domain-specific view of WM (Waters and Caplan, 1996).

While the effect of WM on the N400-P600 complex elicited by ORCs on the relativizer supports the shared resource view of WM, the absence of such an effect on P600 elicited by SRCs on the head noun supports the dedicated resource account of WM. These results mirror the conflicting findings in the literature, in support of a dedicated resource view (Caplan and Waters, 1999, 2013; Waters and Caplan, 2005), and of the shared resource view (Just and Carpenter, 1992; Daneman and Merikle, 1996; Keller et al., 2001; Just et al., 2003). Although seemingly contradictory, the findings can be reconciled if we assume that the domain-general verbal WM system is utilized for maintenance (of multiple interpretations in parsing on the basis of lexical information) and reanalysis processes indexed by N400 and P600, respectively, and a domain-specific, syntactic specialization in the verbal WM system for processes of integration (integration of dependencies), indexed by P600. Obviously, more research is needed to better understand the nature of individual differences in WM and its effect on sentence processing (Lewis et al., 2006).

The present findings pose a challenge to the Structural Distance Hypothesis (SDH, O'Grady et al., 2003) and other structural hypotheses that predict SRC preference for all languages of the world (Keenan and Comrie, 1977). In SDH, structural distance depends on the amount of syntactic nodes/projections in the syntactic tree that intervene between the head noun and the gap. Although syntactic processes may still apply in RC processing, especially in terms of movement and case-marking (Chomsky, 1981), the extent to which these processes are exhibited in psychological behavior is controversial (Bulut and Wu, 2016). Although the N400 and P600 effects elicited by ORCs on the relativizer seem to support the prediction of ORC difficulty made by SDH, these effects are likely associated with syntactic reanalysis, rather than a difficulty involving relative clause extraction from the object site, which would have been manifested on the head noun, which is the element extracted and relativized. Therefore, we argue that the weight of integration resources based on linear distance between dependencies is heavier than that of a postulated difficulty associated with relative clause extraction from the object site.

The current findings partially support the frequency-based account (Reali and Christiansen, 2007). As indicated above, previous corpus studies generally indicated that SRCs are more frequent than ORCs in Mandarin Chinese (Hsiao and Gibson, 2003; Pu, 2007; Vasishth et al., 2013). Therefore, SRC advantage is predicted by the frequency-based account, which was not supported by the present finding of P600 on the SRC head noun. However, frequency approaches formalized as experience and surprisal (Hale, 2001; Levy, 2008) and entropy (Hale, 2003) as well as constraint-based approaches (McRae et al., 1998; Reali and Christiansen, 2007; Gennari and MacDonald, 2008) posit that alternative structural interpretations of a sentence being processed are partially activated based on frequency, plausibility, and expectation. This experience-based approach can explain the reanalysis observed on the relativizer in ORCs. As there were multiple possible readings in ORCs until the relativizer, readers needed to resolve the ambiguity when they encountered the relativizer, leading to an N400-P600 complex for ORCs. However, a purely structural frequency approach cannot explain the current findings on the head noun.

The predictions of the similarity-based account (Gordon et al., 2001; Van Dyke and McElree, 2006) were not tested since the referent types in RCs were not manipulated. However, the current findings are also compatible with the predictions of accounts emphasizing decay and interference (Lewis and Vasishth, 2005; Vasishth et al., 2013). These accounts would predict more difficulty with SRCs than ORCs in Chinese for two main reasons. Firstly, there is decay of the referent from the WM due to longer linear distance between the filler and gap in SRCs than ORCs. This assumption has consequences similar to those of the integration resources metric. Secondly, these accounts would predict more difficulty with Chinese SRCs than ORCs because the object NP intervenes between the RC verb and the subject NP, potentially causing interference.

In terms of neurocognitive models of language processing, the ERP effects observed in the current study can be interpreted within the framework of a single-stream model of language processing emphasizing retrieval and integration, reflected by N400 and P600, respectively (Brouwer et al., 2012, 2017). In this model, N400 is associated with the mental processes concurrent with the retrieval of lexical information from long-term memory. This retrieval process is facilitated by the activation of semantic and syntactic features of the preceding lexical items. The N400 effect elicited by ORCs on the relativizer can be interpreted as retrieval cost due to low cloze probability along the same lines, as the preceding structure (a verb phrase) activates, or primes, the semantic and syntactic features of a noun that should follow and serve as the object of the embedded clause, rather than a relativizer. According to the model, the P600 effect involves “the construction, revision, or updating of a mental representation of what is being communicated” (Brouwer et al., 2012). Accordingly, the P600 elicited by the relativizer in ORCs can be interpreted as a revision of the mental representation that was constructed before (subordinate-clause reading), but turned out not to be the correct one (RC reading). On the other hand, the P600 observed on the head noun can be explained as construction of a coherent representation by establishing a thematic dependency between the head noun and the RC verb.

The present ERP findings do not provide unambiguous evidence for either an SRC preference or difficulty in Chinese. It seems that multiple linguistic operations with potentially opposite processing consequences take place when reading Chinese SRCs and ORCs. These opposing forces in processing of Chinese RCs involving resolution of syntactic ambiguities (on the relativizer), and integration of filler-gap dependencies (on the head noun) might lead to opposite processing preferences at different locations in Chinese RCs, as reflected in the present study. These opposing factors might underlie the conflicting findings reported in the literature, as outlined in Table 1, and also the lack of a clear SRC preference for Chinese in contrast with the findings in Indo-European languages.

## Conclusion

The present study used ERPs together with two tests of working memory (WM) span to investigate processing of Chinese subject and object relative clauses and potential effects of WM capacity on processing patterns. The results revealed an N400-P600 complex elicited by ORCs on the relativizer, which was interpreted as indicative of syntactic reanalysis. A P600 effect was elicited by Chinese SRCs on the head noun region for both high and low WM span groups. This finding supports a sentence processing account based on integration resources which are tasked when the linear distance between dependencies is long. As the scores of WM span obtained by the forward digit span and operation span tests interacted with the N400-P600 complex on the relativizer, but did not modulate the P600 effect on the head noun, both domain-general and domain-specific aspects of verbal WM resources are proposed. These findings demonstrate a complex relative clause processing pattern for Chinese without a clear preference for SRC or ORC, in which opposing factors including structural ambiguities and integration of filler-gap dependencies alter processing dynamics.

## Author contributions

TB was involved in designing, planning and conducting the experiments, analyzing and interpreting the results, writing up and editing the manuscript. S-KC was involved in designing, planning and conducting the experiments, interpreting the results and editing the manuscript. K-YX was Involved in interpreting the results and editing the manuscript. DH was involved in interpreting the results and editing the manuscript. DW was involved in designing, planning and conducting the experiments, analyzing and interpreting the results, writing up and editing the manuscript.

### Conflict of interest statement

The authors declare that the research was conducted in the absence of any commercial or financial relationships that could be construed as a potential conflict of interest.
