# Supplementary material for: Is There a Processing Preference for Object Relative Clauses in Chinese? Evidence From ERPs
Source: Front Psychol. 2018 Jul 9;9:995. doi: 10.3389/fpsyg.2018.00995 (PMC6046449; doi:10.3389/fpsyg.2018.00995)
Supplement: Supplementary file 1 [file Data_Sheet_1.DOCX]

Supplementary Material

Is there a processing preference for object relative clauses in Chinese? Evidence from ERPs

Talat Bulut, Shih-Kuen Cheng, Kun-Yu Xu, Daisy L. Hung, Denise H. Wu*

*** Correspondence:** Corresponding Author: denisewu@cc.ncu.edu.tw

# Supplementary Data

- 1. **Analyses on Relative Clause Verb and Noun**

In Chinese relative clauses, the embedded verb and noun are ordered differently in SRCs and ORCs; that is, SRCs have a verb followed by a noun, while ORCs have a noun followed by a verb in this region. This difference complicates their comparison, as different lexical items have to be compared, which may elicit ERP effects tied to lexical differences (verb versus noun). Nevertheless, in order to paint a complete picture of ERP correlates of relative clause processing, we analyzed the ERPs on the relative clause embedded verb and noun and the results are given below.

The same preprocessing steps as reported in the main article were applied, and ERPs were computed for a single, multiword epoch of 1400 ms comprising the SRC embedded verb / ORC embedded noun (Word 1) and the SRC embedded noun / ORC embedded verb (Word 2) with a 200-ms prestimulus baseline. The same statistical procedures as in the main article were carried out. Statistical tests were conducted on ERPs for the medial (Fz, Cz, Pz) and lateral electrodes (F7-F8, F3-F4, C5-C6, P3-P4, T7-T8). A 100-ms moving window analysis was conducted on the ERPs.

At the artifact rejection step, three participants were removed from the analysis due to excessive loss of trials. The remaining participants contributed an average of 63.5% of the trials to the analysis [number of accepted trials: SRC, Mean=25, Range=12-40; ORC, Mean=26, Range=11-39]. The number of rejected trials did not differ statistically across conditions (*t*(20)=1.369, *p*=.186). As in the main article, the forward digit span and operation span scores were used to assign the participants to the low (N=10) and high (N=11) working memory groups. The operation span and forward digit span scores were proportionally similar within the WM groups [Low WM Group: Operation Span (Mean=.79; SD=.12) & Forward Digit Span (Mean=8.0; SD=1.0); High WM Group: Operation Span (Mean=.91; SD=.03) & Forward Digit Span (Mean=10.64; SD=.77)].

**1.1.1. Results**

Supplementary Tables 1 and 2 summarize the ANOVA *F*-values for the main effect of RC-Type and its interactions with the other factors on Word 1 (SRC verb / ORC noun) and Word 2 (SRC noun / ORC verb), respectively. As illustrated in Supplementary Figure 1, a number of ERP effects were identified, some of which exhibited differences between the WM groups, as reported in detail below.

**Supplementary Table 1.** Summary of ANOVAs on Word 1 (SRC embedded verb / ORC embedded noun).

| **Source** |  | ***F* Values** | | | | | | |
| --- | --- | --- | --- | --- | --- | --- | --- | --- |
| **Medial ANOVA** | df | 0-100ms | 100-200ms | 200-300ms | 300-400ms | 400-500ms | 500-600ms | 600-700ms |
| RC-Type | 1,19 | 0.073 | 0.359 | 1.406 | 1.527 | 0.009 | 0.117 | 0.027 |
| RC-Type x Electrodes | 2,38 | 2.664 | 3.038 | **11.189**** | **4.450*** | 1.385 | 0.521 | 0.053 |
| RC-Type x WM | 1,18 | 1.737 | 1.659 | **16.622***** | 0.997 | 2.733 | 0.896 | 0.729 |
| RC-Type x Electrodes x WM | 2,36 | 0.299 | 0.134 | 0.284 | 0.683 | 0.072 | 0.216 | 0.045 |
| **Lateral ANOVA** |  |  |  |  |  |  |  |  |
| RC-Type | 1,19 | 0.060 | 1.621 | **6.515*** | 0.322 | 0.086 | 0.001 | 0.151 |
| RC-Type x Electrodes | 4,76 | 0.361 | 1.521 | **7.315**** | 2.010 | 0.201 | 0.043 | 0.621 |
| RC-Type x Hemisphere | 1,19 | 1.580 | 1.240 | 0.002 | 0.001 | 1.110 | 3.038 | 0.108 |
| RC-Type x Hemisphere x Electrodes | 4,76 | 0.944 | 1.208 | 0.752 | 0.873 | 0.461 | 1.059 | 1.138 |
| RC-Type x WM | 1,18 | 1.301 | 0.940 | **5.285*** | 0.115 | 0.551 | 0.437 | 0.304 |
| RC-Type x Electrodes x WM | 4,74 | 2.338 | 1.028 | 2.181 | 0.547 | 1.213 | 0.844 | 0.820 |
| RC-Type x Hemisphere x WM | 1,17 | **5.511*** | 0.217 | 2.681 | 0.995 | 0.765 | 0.324 | 0.919 |
| RC-Type x Hemisphere x Electrodes x WM | 4,72 | 0.427 | 0.090 | 1.084 | 0.286 | 0.369 | 0.382 | 0.376 |

* p<0.050; ** p≤0.010; *** p≤0.001

**Supplementary Table 2.** Summary of ANOVAs on Word 2 (SRC embedded noun / ORC embedded verb).

| **Source** |  | ***F* Values** | | | | | | |
| --- | --- | --- | --- | --- | --- | --- | --- | --- |
| **Medial ANOVA** | df | 700-800ms | 800-900ms | 900-1000ms | 1000-1100ms | 1100-1200ms | 1200-1300ms | 1300-1400ms |
| RC-Type | 1,19 | 0.146 | 0.582 | 1.527 | 0.117 | 1.581 | 0.303 | 0.169 |
| RC-Type x Electrodes | 2,38 | 0.054 | 0.130 | 2.827 | **3.663†** | 0.646 | 0.473 | 0.363 |
| RC-Type x WM | 1,18 | 0.589 | 0.486 | 0.190 | 0.409 | 3.237 | 0.122 | 0.110 |
| RC-Type x Electrodes x WM | 2,36 | 0.118 | 0.035 | 0.067 | 0.062 | 0.066 | 0.126 | 0.012 |
| **Lateral ANOVA** |  |  |  |  |  |  |  |  |
| RC-Type | 1,19 | 0.214 | 0.253 | 3.611 | 0.962 | 2.221 | 0.758 | 0.383 |
| RC-Type x Electrodes | 4,76 | 0.672 | 0.979 | **3.785*** | **3.180*** | 1.329 | 0.511 | 0.424 |
| RC-Type x Hemisphere | 1,19 | 0.222 | 0.007 | 0.191 | 2.200 | 3.338 | 0.587 | 0.595 |
| RC-Type x Hemisphere x Electrodes | 4,76 | 1.781 | 1.598 | 2.323 | **2.595†** | 2.399 | 2.345 | 1.289 |
| RC-Type x WM | 1,18 | 0.219 | 0.129 | 0.251 | 0.064 | 0.516 | 0.026 | 0.001 |
| RC-Type x Electrodes x WM | 4,74 | 1.070 | 0.988 | 1.104 | 1.109 | 1.138 | 1.388 | 1.293 |
| RC-Type x Hemisphere x WM | 1,17 | 0.462 | 0.702 | 0.325 | 0.021 | 2.731 | 2.502 | 1.289 |
| RC-Type x Hemisphere x Electrodes x WM | 4,72 | 0.391 | 0.504 | 0.340 | 0.241 | 0.518 | 0.616 | 0.911 |

**†** p≤.056; * p<0.050

**A. Low WM Group**

**
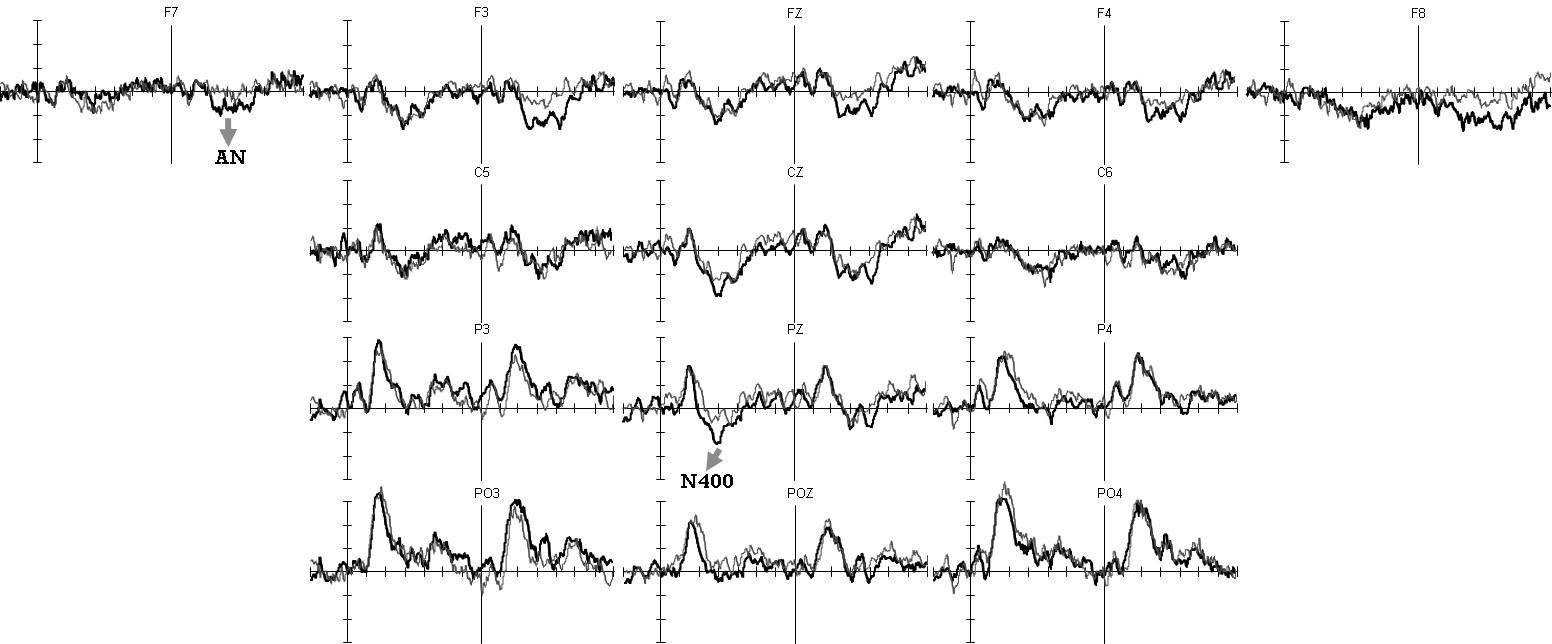
**

**B. High WM Group**

**
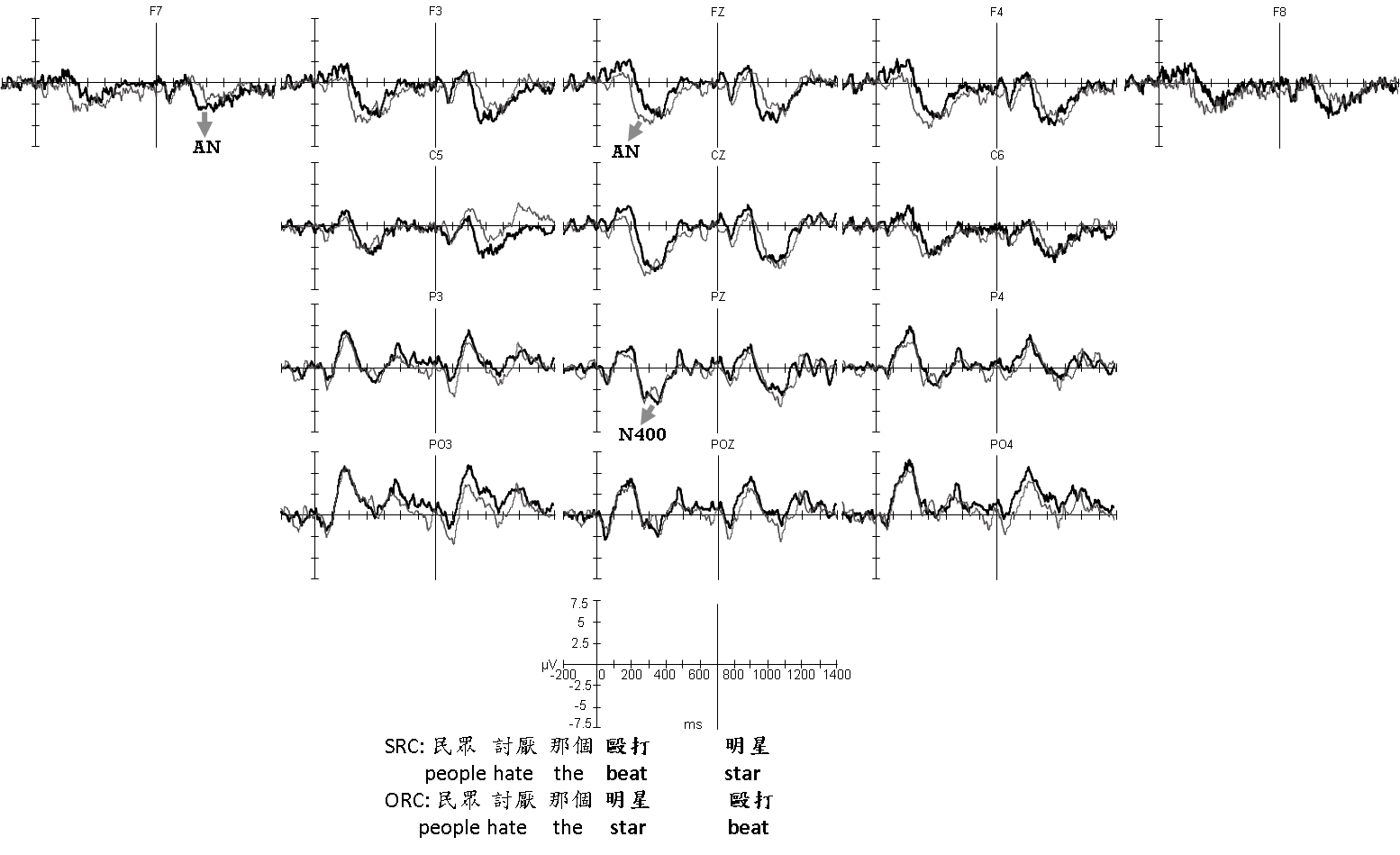
**

**C.**

**
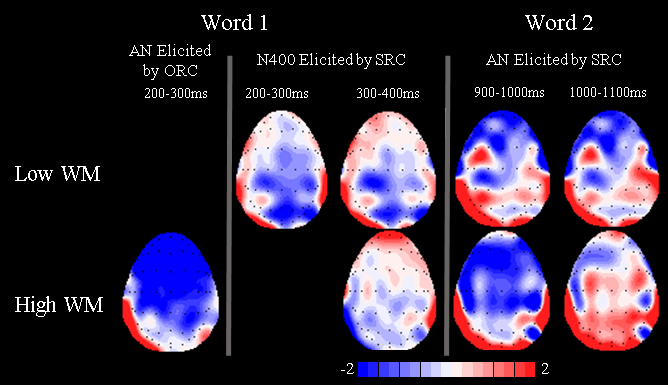
**

**Supplementary Figure 1.** Grand average ERPs on the relative clause verb and noun for the low working memory group (Panel A) and the high working memory group (Panel B). Topographic distributions of the difference waves showing the ERP effects elicited by SRC and ORC on Word 1 (SRC verb / ORC noun) and Word 2 (SRC noun / ORC verb) for the low and high working memory groups (Panel C).

- - - 1. **ERPs on the First Embedded Word**

The first embedded word (Word 1) corresponded to the embedded verb in SRCs, and the embedded noun in ORCs. A significant interaction of RC-Type*Hemisphere*WM was found in the first time window (0-100ms) in the lateral ANOVA [(*F*(1,17)=5.511, *p*=.030, η^2^=.225]. This interaction was followed up with step-down ANOVAs, which did not reveal any significant effects [(*F*s≤2.670, *p*s≥.133].

Visual inspection of ERPs at frontal electrodes in Supplementary Figure 1 suggests that there is a negativity elicited by ORCs for the high WM group between 200-300 ms. This negativity was evidenced by a significant main effect of RC-Type [(*F*(1,19)=6.515, *p*=.019, η^2^=.255], and significant interactions of RC-Type*WM [(*F*(1,18)=5.285, *p*=.033, η^2^=.218] and RC-Type*Electrodes [(*F*(4,76)=7.315, *p*=.002, η^2^=.278] in the lateral ANOVA, and significant interactions of RC-Type*WM [(*F*(1,18)=16.622, *p*=.001, η^2^=.467] and RC-Type*Electrodes [(*F*(2,38)=11.189, *p*=.002, η^2^=.371] in the medial ANOVA between 200-300 ms. Follow-up analyses revealed that ORCs elicited more negativity than SRCs at frontal electrodes (F7-F8, F3-F4, FZ) [(*F*s(1,10) ≥11.020, *p*s≤.008, η^2^s≥.524], central electrodes (C5-C6, CZ) [(*F*s(1,10)≥12.107, *p*s≤.006, η^2^s≥.548] and temporal electrodes (T7-T8) [(*F*(1,10) =6.509, *p*=.029, η^2^=.394] only for the high WM group, while there was no significant difference between SRCs and ORCs at these electrodes for the low WM group [(*F*s(1,9)≤3.544, *ps*≥.092]. This negativity elicited by the ORC embedded noun when compared to the SRC embedded verb only for the high WM group resembles an anterior negativity due to its latency and topographic distribution, as illustrated in Supplementary Figure 1(C).

The follow-up analyses in the time window of 200-300 ms also showed that SRCs elicited more negativity than ORC at PZ for the low WM group [(*F*(1,9) =7.675, *p*=.022, η^2^=.460], while there was no significant difference at PZ for the high WM group [(*F*(1,10)=1.227, *p*=.294, η^2^=.109]. This negativity extended into the following time window (300-400 ms), where it was significant for both WM groups. Specifically, the medial ANOVA revealed a significant interaction of RC-Type*Electrodes between 300-400 ms [(*F*(2,38)=4.450, *p*=.034, η^2^=.190]. Step-down ANOVAs showed that this interaction stemmed from significantly greater negativity elicited by SRCs than ORCs at PZ, while there was no significant difference at other medial electrodes [(*F*s(1,20)≤1.709, *p*s≥.206]. This negativity, which was elicited by the SRC embedded verb when compared to the ORC embedded noun, resembles an N400 effect due to its latency and scalp distribution, as illustrated in Supplementary Figure 1(C), and likely involves lexical differences between the conditions (verb versus noun). Interestingly, it had an earlier onset for the low WM group (extending from 200 ms to 400 ms), while it was significant only between 300-400 ms for the high WM group.

Finally, SRCs elicited a frontal negativity between 900-1100 ms, which corresponds to 200-400 ms following the onset of Word 2 (SRC embedded noun / ORC embedded verb). This effect was reflected in the significant interaction of RC-Type*Electrodes in the lateral ANOVA in both the 900-1000 ms time window [(*F*(4,76)=3.785, *p*=.021, η^2^=.166] and the 1000-1100 ms time window [(*F*(4,76)=3.180, *p*=.039, η^2^=.143]. The step-down ANOVAs revealed that this effect was due to significantly more negativity elicited by SRCs than ORCs at frontal (F7-F8, F3-F4) [(*F*s(1,20)≥6.626, *p*s≤.018, η^2^s≥.249] and temporal electrodes (T7-T8) [(*F*(1,20)=4.675, *p*=.043, η^2^=.189] between 900-1000 ms, and at the frontal electrode pair F7-F8 between 1000-1100 ms [(*F*(1,20)=6.142, *p*=.022, η^2^=.235], while there was no significant difference in any one of the other lateral electrodes [(*F*s(1,20)≤2.439, *p*s≥.134]. The latency and topographic distribution of this negativity illustrated in Supplementary Figure 1(C) suggest that it might be an anterior negativity elicited by the SRC embedded noun when compared to the ORC embedded verb.

In summary, the analyses of ERPs on the relative clause embedded verb and noun revealed an anterior negativity elicited by ORCs on Word 1 (ORC noun / SRC verb) for the high WM group only, whereas SRCs elicited an N400 effect on the same word for both WM groups (with an earlier onset in the low WM group). On the other hand, there was an anterior negativity elicited by SRCs on Word 2 (SRC noun / ORC verb) for both WM groups.

- 1. **Initial Analysis on Head Noun with a Problematic Baseline**

As stated in the main article, we initially analyzed the ERPs on the head noun as a separate 700-ms time window with a 100-ms prestimulus baseline, which corresponds to the period between 600-700 ms after presentation of the relativizer, as shown in Supplementary Figure 2A. However, because part of the P600 effect elicited by ORCs on the relativizer corresponded to this baseline period adopted for the head noun (as illustrated in Figure 2 in the main article), this not only led to cutting off the ongoing P600 effect on the relativizer, but also introduced a confound in the ERPs on the head noun, such that the P600 effect elicited by SRCs on the head noun was exaggerated (as discussed in Steinhauer and Drury, 2012). In other words, as the ERPs for ORCs were more positive than those for SRCs during the potentially problematic baseline, ORC waveform on the head noun was artificially shifted towards the negative polarity, creating at least part of the positivity for SRCs on the head noun.

Statistical tests for ERP data on the head noun were run using a series of repeated-measures ANOVAs for each 50-ms time window. The ANOVAs were conducted with three within-subject factors (RC type, laterality, caudality) and one between-subjects factor (WM group).The RC type factor consists of subject and object relative clause conditions. The laterality and caudality factors corresponded to electrode sites, each comprising three levels (laterality: left (L), mid (M), right (R); caudality: anterior (A), central (C), posterior (P)). Thus, signals were collected from nine sites comprising the following electrodes: LA: F7,F5,F3,FT7,FC5,FC3; MA: F1,FZ,F2,FC1,FCZ,FC2; RA: F4,F6,F8,FC4,FC6,FT8; LC: T7,C5,C3,TP7,CP5,CP3; MC: C1,CZ,C2,CP1,CPZ,CP2; RC: C4,C6,T8,CP4,CP6,TP8; LP: P7,P5,P3,PO7,PO5,O1; MP: P1,PZ,P2,PO3,POZ,PO4,OZ; RP: P4,P6,P8,PO6,PO8,O2. Greenhouse-Geisser correction was applied for deviations from sphericity in the data. The ANOVA *F* values and the ERPs with topographic distributions pertaining to this initial analysis are given in Supplementary Table 3 and Supplementary Figure 2, respectively. As illustrated in Supplementary Figure 2, there was a posterior negativity associated with SRCs, which prevailed almost throughout the entire analysis time frame, as evidenced by the significant interaction of RC-Type*Caudality in Supplementary Table 3.

**Supplementary Table 3.** Summary of ANOVAs on the head noun analyzed with the problematic baseline.

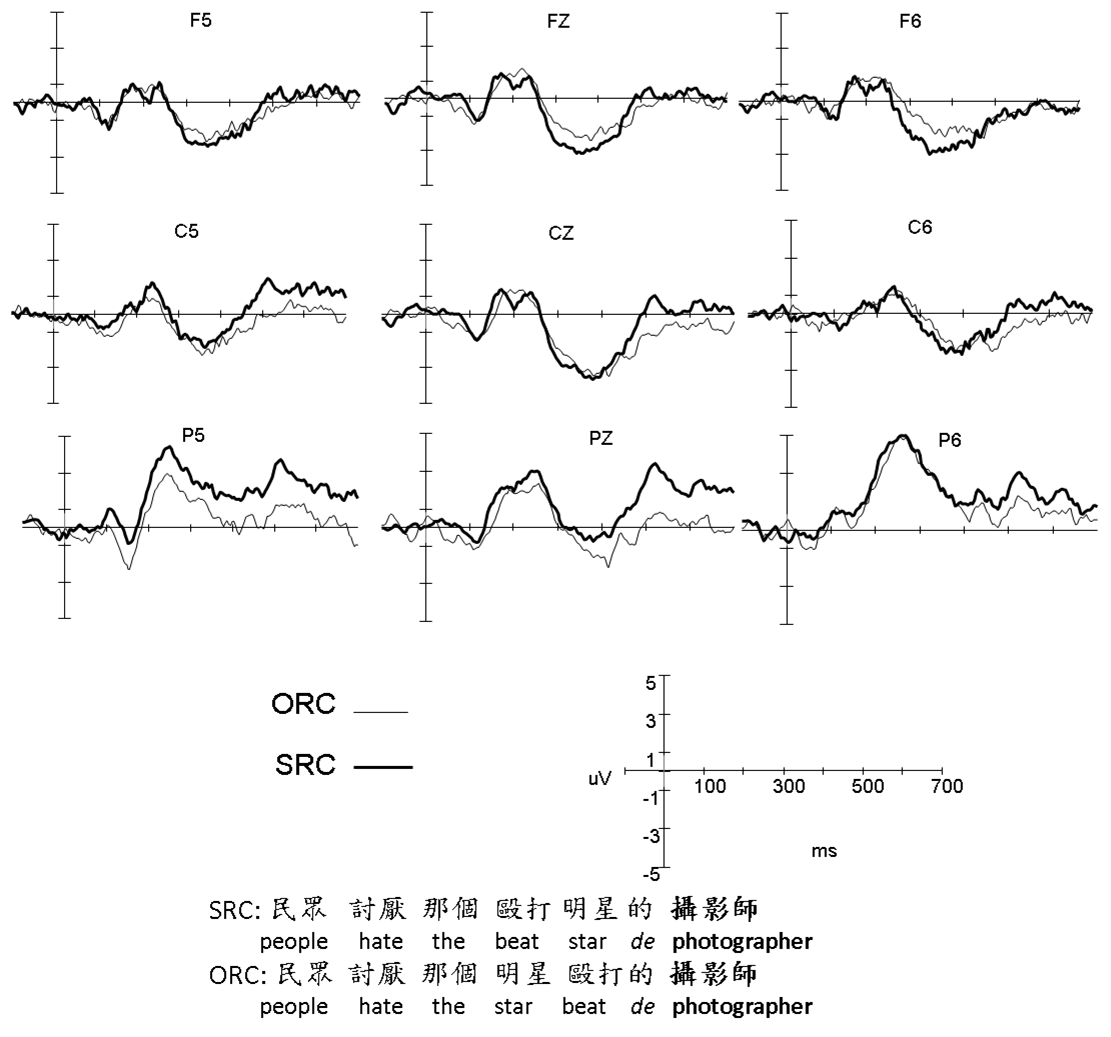
**A.**

**B.**

**Supplementary Figure 2.** Grand average ERPs on the head noun (A) and topographic distribution of the difference waves (calculated by subtracting the ORC wave from the SRC wave) (B), as analyzed with the problematic baseline.

**
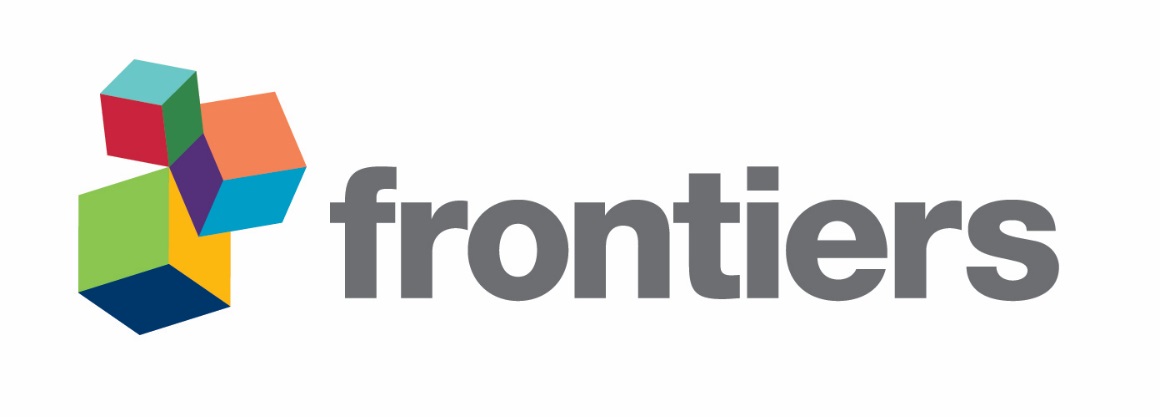
**
